# FESW-UNet: A Dual-Domain Attention Network for Sorghum Aphid Segmentation

**DOI:** 10.3390/s26020458

**Published:** 2026-01-09

**Authors:** Caijian Hua, Fangjun Ren

**Affiliations:** School of Computer Science and Engineering, Sichuan University of Science and Engineering, Yibin 644000, China; 323085406121@stu.suse.edu.cn

**Keywords:** sorghum aphids, image segmentation, FESW-UNet, dual-domain attention, pest monitoring

## Abstract

Current management strategies for sorghum aphids heavily rely on indiscriminate chemical application, leading to severe environmental consequences and impacting food safety. While precision spraying offers a viable remediation for pesticide overuse, its effectiveness depends on accurately locating and classifying pests. To address the critical challenge of segmenting small, swarming aphids in complex field environments, we propose FESW-UNet, a dual-domain attention network that integrates Fourier-enhanced attention, spatial attention, and wavelet-based downsampling into a UNet backbone. We introduce an efficient multi-scale attention (EMA) module between the encoder and decoder to enhance global context perception, enabling the model to capture more accurate relationships between global and local features in the field. In the feature extraction stage, we embed a simple attention module (SimAM) to target key infestation regions while suppressing background noise, thereby enhancing pixel-level discrimination. Furthermore, we replace conventional downsampling with Haar wavelet downsampling (HWD) to reduce resolution while preserving structural edge details. Finally, a Fourier-enhanced attention module (FEAM) is added to the skip-connection layers. By using complex-valued weights to regulate frequency-domain features, FEAM effectively fuses global low-frequency structures with local high-frequency details, thereby enhancing feature representation diversity. Experiments on the Aphid Cluster Segmentation dataset demonstrate that FESW-UNet outperforms other models, achieving an mIoU of 68.76%, mPA of 78.19%, and mF1 of 79.01%. The model also demonstrated strong adaptability on the AphidSeg-Sorghum dataset, achieving an mIoU of 81.22%, mPA of 87.97%, and mF1 of 88.60%. The proposed method offers an efficient and feasible technical solution for monitoring and controlling sorghum aphids through image segmentation, demonstrating broad application potential.

## 1. Introduction

Sorghum aphids are one of the major insect pests infesting sorghum in its growth process. Their feeding activity causes direct tissue damage, and the viruses they transmit can further aggravate yield reduction, collectively posing a serious threat to both crop productivity and quality. Currently, chemical pesticides remain the dominant strategy for the management of sorghum pests, diseases, and weeds. Studies have shown that pesticide use can help recover approximately 20–25% of the global annual crop yield that would otherwise be lost [1]. Nevertheless, excessive pesticide spraying is not only a cause of the collection of chemical residues in crops but also other forms of pollution to the environment. This highlights the urgent need for precision spraying technology, which enables the precise application of pesticides by accurately detecting pest species and their spatial distribution. This approach can effectively reduce pesticide pollution, significantly improve spraying efficiency, and minimize pesticide waste.

In order to detect pests in crop production, various methods have been developed [2,3], which can be broadly categorized into machine learning approaches [4] and deep learning techniques [5,6]. Segmentation is an essential aspect in the pest image analysis because it enables the precise localization of insect body regions. Among traditional image processing approaches, Kumar et al. [7] proposed a computationally efficient detection algorithm combining median filtering with adaptive threshold segmentation. By optimizing the denoising and segmentation workflow, it achieved over 95% accuracy in complex backgrounds. Zhang et al. [8] applied a composite gradient watershed algorithm in pest images segmentation, demonstrating lower relative errors compared to conventional methods. Subsequently, Deng et al. [9] enhanced image segmentation for corn pest detection by integrating an improved GrabCut algorithm with saliency and depth information, achieving high accuracy with minimal manual intervention. Although these approaches are reported to perform well in specific settings, they generally have manual parameter adjustment needs as well as low generalizability when exposed to pest image collections that are more varied. Specifically, they find it challenging to bridge the semantic gap between low-level pixel intensity and high-level biological identity in crowded scenes.

The rapid advancement of deep learning has primarily surpassed traditional machine learning in feature extraction, resulting in significant breakthroughs in pest detection. Numerous CNN-based studies have achieved high accuracy, ranging from general detectors like YOLO [10], SSD [11], and Faster R-CNN [12], to specialized models such as PestNet [13] and DeepPest [14]. Other approaches have further optimized performance through sampling-balanced RPNs [15] or by integrating heterogeneous data sources, such as meteorological variables [16]. However, these methods primarily focus on object detection. Although rough boundary frames are effective for distinct objects, they are far from enough for pests that form dense overlapping communities. In order to accurately quantify the severity of pests and biomass in such cases, semantic segmentation is crucial to providing accurate pixel-level boundary division.

With the rapid advancement of semantic segmentation techniques, pixel-level pest identification has increasingly been addressed within a semantic segmentation network. Zhao et al. [17] leveraged transfer learning to achieve instance segmentation, while Shen et al. [18] developed an enhanced deep learning based system that supports efficient pest monitoring in grain storage through data augmentation and model refinement. Nevertheless, Kumar et al. [19] identified the constrained receptive fields in their CNN architecture as a fundamental limitation for capturing long-range dependencies within leaf lesion patterns. Although Vision Transformers have demonstrated strong global representation capabilities in many vision tasks [20], their quadratic computational complexity and lack of inductive biases make them challenging to deploy on edge devices with limited resources, such as agricultural drones and IoT terminals, especially for high-resolution imagery captured in field conditions. These constraints highlight the need for representations that go beyond purely spatial modeling. On the other hand, some research has shown that global self-attention primarily focuses on the low-frequency components of the image, potentially neglecting high-frequency information to some extent [21]. In practice, aphid images often contain shadow occlusions, low contrast regions between aphids and leaf surfaces, and blurred boundaries caused by complex textures and illumination variations. These characteristics are typically manifested in the high-frequency components of the frequency domain [22]. Conventional spatial domain methods struggle to represent frequency-sensitive features effectively in the traditional spatial domain. Since frequency domain information is susceptible to intensity variations, its proper utilization can significantly enhance a model’s ability to perceive object boundaries and fine-grained details of aphids.

In recent years, Yu et al. [23] proposed the Frequency Enhanced Saliency Network (FESNet), which utilizes frequency domain cues and spatial domain information to enhance the ability to detect small insects in complex grain storage backgrounds. Ma et al. [24] also developed a similar method. Similarly, Zhang et al. [25] proposed a frequency domain attention network (FdaNet) based on adaptive learning frequency weights, which significantly improved the detection ability of small spotted targets on citrus leaves. However, using only frequency domain features may lose spatial location information, as spatial location information contains rich semantic contexts that are crucial for accurate positioning. In addition, the high degree of visual similarity between pests and host plants also highlights the urgent need to obtain comprehensive semantic information [26].

To this end, we have proposed FESW-UNet. This dual-domain attention network combines frequency-domain clues with spatial attention mechanisms to address the limitations of relying solely on single frequency-domain information. The architecture builds an integration of components on the UNet backbone. Particularly, we utilize the EMA module to enhance semantic representations and thus make them resistant to variations in the scale of field scenarios. At the same time, we also utilize the SimAM and a hybrid loss design, which enhances the saliency of aphid regions and converges more quickly. To achieve the best feature compression, HWD is employed instead of the usual operations, allowing for the preservation of boundary information in high frequencies and minimizing certain computations. Finishing the architecture, the FEAM combines spatial architecture with frequency-domain features, which is an effective way of offsetting the effects of uniform illumination and jagged background interference. This research aims to achieve the following main goals:

- Build a dual-domain attention network, FESW-UNet, based on UNet to overcome the characterization limitations of existing methods that only rely on single-frequency domain information, so that it can improve segmentation performance while maintaining high reasoning efficiency.
- Design the FEAM and insert it into the skip connections of UNet to jointly use spatial structure information and frequency texture data to distinguish aphid areas in complex backgrounds.
- Build the sorghum aphid dataset AphidSeg-Sorghum and evaluate the flexibility and performance of FESW-UNet in different collection environments and data domains.

## 2. Materials

**Figure 1:**
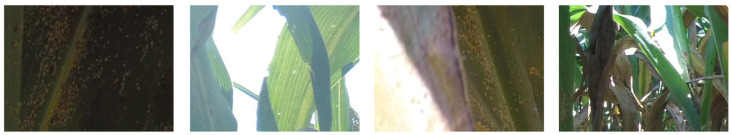
Representative images of sorghum aphids collected under complex field conditions from the Aphid Cluster Segmentation dataset.

In this study, the Aphid Cluster Segmentation dataset, published by Rahman et al. [27], was initially employed as the primary experimental dataset. Before use, the image goes through a series of preprocessing steps, including quality screening, removing out-of-focus samples, and eliminating unavailable photos. After cleaning, 7720 high-quality aphid images were retained. The dataset provides sorghum leaf photos collected under diverse real-world field conditions, incorporating a wide range of illumination types, background textures, and multiple degrees of aphid aggregation and infestation. Figure 1 shows some examples of sorghum aphid images in the Aphid Cluster Segmentation dataset, which were collected under complex field conditions. These scenes exhibit significant differences in light intensity or weakness (as seen in the first three images), the degree of background disorder (as illustrated in the fourth image), aphid density and spatial distribution (as observed in the third image), and the geometry of the leaves. These examples illustrate the practical challenges faced by segmentation models and the need to build a robust architecture that can handle small targets and heterogeneous agricultural environments.

**Figure 2:**
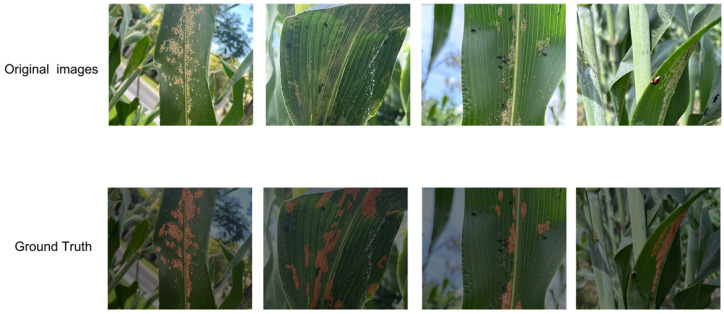
Sample images and corresponding pixel-level annotations from the AphidSeg-Sorghum dataset.

In addition to the above reference dataset, we have also created a sorghum aphid image dataset, called AphidSeg-Sorghum, to test further the model’s performance in other data acquisition and image environments. The photos were taken at the Experimental Laboratory of the Engineering Research Center for Special Sorghum to Liquor Production of Sichuan University of Science and Engineering, as well as at the Wuliangye sorghum planting base. Mobile phones were the imaging equipment that allowed for gathering multi-angle and multi-scale samples of infested areas under diverse lighting conditions, time intervals, perspectives, and distances to imitate real-life field monitoring situations. During the supervised learning process, we use the LabelMe tool (version 5.6.0) to create pixel-level annotations manually, ensuring the correctness and consistency of the labels. Figure 2 below indicates the regions of the sorghum aphids in red. The number of original images left after cleaning was 612. To build the expanded dataset used in the experiment, we randomly divided the 612 images into four subsets of equal size and non-overlapping (each subset contains a quarter of the data). For the four subsets, we generated one additional sample per image by applying a single transformation from the following operations, respectively: horizontal flipping, vertical flipping, brightness perturbation, and noise injection. The result is that we are introduced to 612 extra images, bringing the total size of the resultant AphidSeg-Sorghum dataset to 1224 and presented in Table 1 dataset (one per four subsets): The expansion by two doubles without introducing a large ratio of simulated information but a rational mixture of viewpoint (flip) and imaging-condition (brightness and noise) alterations that depict field monitoring and will enhance the robustness and external domain generalization.

A concise summary of the two datasets is provided in Table 1. All data were partitioned into training and testing subsets using a 7:3 ratio to maintain both sufficient training samples and representative evaluation data.

## 3. Methods

### 3.1. Overall Architecture of FESW-UNet

**Figure 3:**
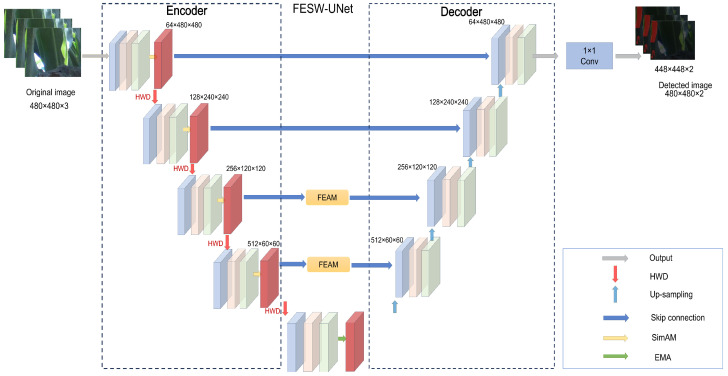
Overall architecture of the proposed FESW-UNet. Different colored squares denote feature maps at different scales. Colors are used only for visual clarity.

As presented in Figure 3, the architecture of FESW-UNet is based on the conventional UNet encoder–decoder and includes modules addressing four particular tasks, thus creating a successive process of feature optimization. In the encoding stage, we embed the SimAM module to provide a parameterless pixel-level attention mechanism, which can amplify the unique signals of aphids while actively suppressing environmental noise, such as leaf veins, reflections and shadows. To mitigate the loss of spatial details caused by stride-2 convolutions, we adopt the HWD module for downsampling. It uses Haar wavelets to decompose features into frequency bands, preserving edge information while reducing computational cost. At the network bottleneck, the feature diagram is rich in semantics. We inserted an EMA module to adaptively integrate multi-scale context information, thereby strengthening the connection between the global structure and local details to accommodate significant changes in pest scale. At this final step, we utilize the FEAM to optimize the nature of the skip connection. FEAM adjusts low-frequency content and high-frequency content across complex-valued weights in the frequency domain and combines them again into the spatial domain. The collaborative design is effective in balancing both spatial fidelity and spectral analysis and therefore attains good feature learning and high segmentation accuracy in a dynamic agricultural setting.

### 3.2. EMA Module

**Figure 4:**
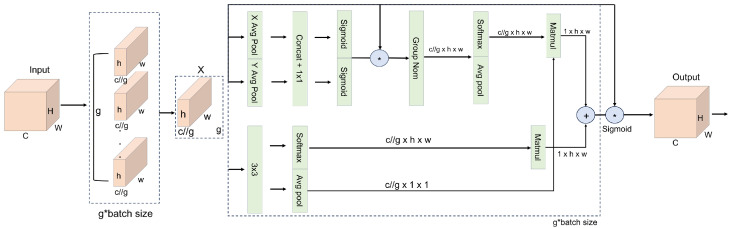
Overall structural diagram of EMA. “*” indicates element-wise multiplication, and “+” indicates element-wise addition. “...” indicates repeated identical elements. Colors are used only for visual clarity.

The EMA module [28] computes multi-scale attention and jointly captures global and local contextual cues, enabling the network to consistently focus on aphid-related features even under complex field conditions (e.g., varying illumination, strong leaf-texture patterns, and curled leaf surfaces), as shown in Figure 4. In this module, XAvgPool and YAvgPool denote one-dimensional global average pooling operations along the horizontal (width) and vertical (height) directions, respectively, and Re-weight represents a channel-recalibration vector. For an input feature map $A\in R^{C\times H\times W}$, EMA partitions the *C* channels into *g* groups, with each group learning complementary semantic cues (e.g., separating leaf-vein structures from aphid clusters or distinguishing dense aphid aggregations from sparse distributions). This grouping design, together with directional pooling, helps suppress structured background interference (such as soil, leaf veins, or specular reflections) while enhancing the contrast between aphid regions and their surroundings. By jointly leveraging grouped features and anisotropic pooling, EMA strengthens the association between global leaf geometry and local aphid appearance, thereby improving the model’s stability and robustness under varying lighting and scale conditions.

The spatial attention map in EMA is generated by three parallel branches: two 1×1 convolutional pathways and one 3×3 convolutional pathway. The 1×1 branches, combined with XAvgPool and YAvgPool, encode directional information along the horizontal and vertical axes, making the network more sensitive to subtle boundary transitions and helping it better distinguish aphids from strong vein textures or reflective artifacts. The 3×3 branch focuses on local morphological and contextual dependencies that are closely related to aphid clustering. In particular, the 1×1 paths first aggregate responses along each spatial dimension via one-dimensional global pooling, and then incorporate broad spatial context through subsequent two-dimensional pooling, whereas the 3×3 path enriches localized structural details. The outputs of all branches are fused and passed through a Sigmoid function to produce the final spatial attention map, which amplifies fine-grained aphid-related cues while preserving essential spatial structures. In dynamically changing field environments, EMA thus adaptively balances global leaf-level structure with local aphid-level patterns, suppresses illumination- and texture-induced noise, and sharpens weak boundaries, contributing to more reliable aphid extraction and improved overall segmentation accuracy.

### 3.3. SimAM Module

**Figure 5:**
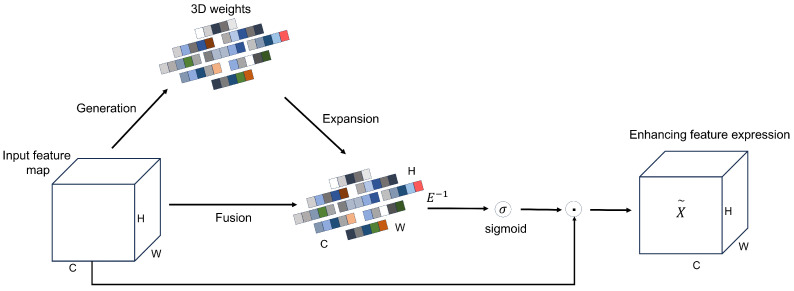
Overall structural diagram of SimAM. The colored squares are a schematic visualization of the generated 3D weight tensor.

Sorghum aphid detection faces challenges due to blurred patterns and low contrast against complex backgrounds. Additionally, practical monitoring systems demand fast inference speeds. We addressed these issues by embedding the SimAM attention module [29] into the feature extraction pipeline (Figure 5). SimAM functions as a parameter-free mechanism that models feature responses to identify key regions automatically. It performs pixel-wise reweighting to emphasize aphid-related cues while suppressing background noise. This refinement promotes more efficient feature propagation and enhances the model’s stability and precision when dealing with complex agricultural field environments, especially in low-contrast scenes with strong leaf-vein or reflection interference.

Given an input feature map of size C×H×W, SimAM estimates a 3D importance map by assigning an energy value to each neuron through an energy function, as defined in Equation (1). The energy for neuron *t* is given by

(1) $$e_{t}\left(w_{t},b_{t},y,x_{i}\right)={\left(y_{t}-\hat{t}\right)}^{2}+\frac{1}{M-1}\sum_{i=1}^{M-1}{\left(y_{0}-{\hat{x}}_{i}\right)}^{2}$$

where $e_{t}$ denotes the energy of neuron *t*, and $w_{t}$ and $b_{t}$ represent the weight and bias of the linear transformation, respectively. The variable $y_{t}$ is the output of neuron *t* on the current channel, *M* denotes the number of neurons in that channel, $y_{0}$ is the output of neighboring neurons, and $x_{i}$ represents the surrounding neuron values (with $\hat{t}$ and ${\hat{x}}_{i}$ denoting their corresponding linear estimates). Intuitively, this formulation penalizes large deviations between the response of neuron *t* and that of its neighborhood, so that neurons with responses very different from their surroundings are assigned lower energy (i.e., higher importance).

Assuming that all pixels within a single channel follow the same distribution, the importance of each independent neuron can be further expressed by the following minimum energy formulation, as given in Equation (2):

(2) $$e_{t}^{*}=\frac{4({\hat{\sigma }}^{2}+\lambda )}{{\left(t-\hat{\mu }\right)}^{2}+2{\hat{\sigma }}^{2}+2\lambda }$$

where $\hat{\mu }=\frac{1}{M}\sum_{i=1}^{M}x_{i}$ and ${\hat{\sigma }}^{2}=\frac{1}{M-1}\sum_{i=1}^{M-1}{\left(x_{i}-\hat{\mu }\right)}^{2}$ denote the mean and variance of all neurons in the channel, and λ is a small regularization term. The value $e_{t}^{*}$ reflects the importance of neuron *t*: a smaller energy corresponds to a larger deviation from its neighborhood and thus a stronger linear separability. Therefore, SimAM adopts $e_{t}^{*}$ as the basis for computing attention weights. Let *E* denote the energy map composed of all $e_{t}^{*}$ values over the feature map; after applying a Sigmoid-based normalization to obtain the attention weights, the enhanced feature map $X^{\prime }$ is computed by element-wise multiplication with the original feature map *X*, as formulated in Equation (3):

(3) $$X^{\prime }=\mathrm{sigmoid}\;\left(\frac{1}{E}\right)⊙X$$

By reweighting features according to the inverse energy map, SimAM suppresses neurons with high energy (low importance) and amplifies those with low energy (high importance). In the context of sorghum aphid segmentation, this mechanism strengthens responses in aphid-infested regions while down-weighting background structures such as leaf veins, glare, and shadows, thereby improving local contrast and boundary clarity without introducing additional learnable parameters or noticeable computational overhead.

### 3.4. HWD Downsampling

**Figure 6:**
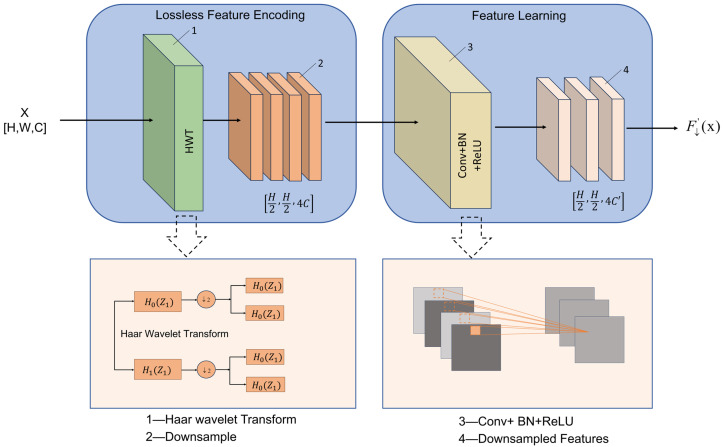
Haar Wavelet Downsampling structure. Solid arrows indicate data flow and dashed arrows indicate the corresponding schematic details. Colors are used only for visual clarity.

In the UNet framework, although traditional maxpooling layers can reduce the resolution of the feature map and highlight the high-response areas, they only retain the maximum activation value in each local window and discard the remaining pixel information. Therefore, essential texture clues, boundary details and structural patterns may be lost, which usually leads to edge blur and spatial distortion. This issue is particularly critical in sorghum aphid segmentation, where aphids in field images are generally small, irregularly distributed, and easily confused with strong leaf-vein textures and complex illumination effects (e.g., shadows and specular reflections). Under such conditions, traditional downsampling operations struggle to maintain a balance between global semantic consistency and the preservation of fine-grained local structural information. To alleviate this problem, this study incorporates the Haar Wavelet Downsampling (HWD) [30] module into the UNet encoder. As illustrated in Figure 6, the HWD module mainly consists of an information-preserving feature-encoding submodule and a feature-representation learning submodule. The feature-encoding component applies the Haar wavelet transform to an input feature map of size H×W with *C* channels, decomposing it into four subbands: an approximation subband (A) and three detail subbands corresponding to horizontal, vertical, and diagonal orientations. By jointly applying the low-pass filter $H_{0}$ and high-pass filter $H_{1}$ along both spatial dimensions, these subbands together perform the downsampling operation, reducing the spatial size of each output feature map to $\frac{H}{2}\times \frac{W}{2}$ while expanding the channel dimension to four times that of the input. This orthogonal spatial–frequency decomposition is theoretically invertible and preserves key structural textures, while still reducing spatial resolution and data redundancy. The one-dimensional Haar basis functions $\delta_{i}\left(x\right)$ and wavelet functions $\psi_{i}\left(x\right)$ used for feature decomposition are defined in Equation (4):

(4) $$\left\{\begin{array}{c} \delta_{1}\left(x\right)=\frac{1}{\sqrt{2}}\varphi_{1,0}\left(x\right)+\frac{1}{\sqrt{2}}\varphi_{1,1}\left(x\right)\\ \psi_{1}\left(x\right)=\frac{1}{\sqrt{2}}\varphi_{1,0}\left(x\right)-\frac{1}{\sqrt{2}}\varphi_{1,1}\left(x\right) \end{array}\right.$$

where the Haar scaling function $\varphi_{j,k}\left(x\right)$ is given by Equation (5):

(5) $$\varphi_{j,k}\left(x\right)=\sqrt{2^{j}}\;\varphi \left(2^{j}x-k\right),\;k=0,1,\ldots ,2^{j}-1$$

with *j* denoting the scale (corresponding to the depth of the convolutional layers in the image domain) and *k* representing the index of the Haar basis function.

The feature-representation learning submodule consists of a standard 1×1 convolution layer, a batch-normalization layer, and a ReLU activation function. In this submodule, the 1×1 convolution is used to maintain compatibility with subsequent layers and to adjust the number of feature channels, while helping to suppress redundant responses and highlight informative components. Adaptive fusion of different frequency bands through reweighting of directional subbands is made possible by parameters that can be learned. Through this mechanism, high-frequency features are preferentially enriched to fix irregular edges in thick aphid patches. At the same time, to achieve a smooth background in the network, low-frequency components are enhanced to retain the global structure and reduce environmental noise. In this multi-level frequency-spatial cooperation during information reduction, the HWD module can improve the compressibility of feature representations, in addition to removing information redundancy, thereby enabling FESW-UNet to achieve greater feature compressibility and structural conservation in environments with complex sorghum fields.

### 3.5. FEAM Module

**Figure 7:**
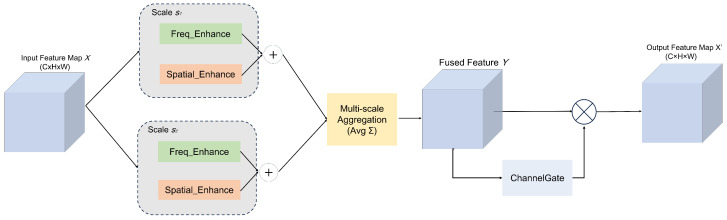
Overall structural diagram of FEAM.

To enhance the real-time segmentation capability for sorghum aphids, this study introduces an improved Fourier-Enhanced Attention Module (FEAM) into the skip-connection layers of UNet. As shown in Figure 7, field images are often affected by diverse factors such as strong or uneven illumination, complex leaf-vein textures, and the coexistence of densely clustered and sparsely distributed aphids. Under these conditions, purely spatial-domain processing or purely frequency-domain processing alone often fails to simultaneously capture both global structural patterns and fine-grained texture details.

Inspired by the spectral gating mechanism proposed in SpectFormer [31], FEAM adopts a dual-domain, multi-scale collaborative strategy: complex-valued weights are learned at a set of predefined spatial resolutions (e.g., 30×30 and 60×60) and projected to the current feature-map scale, after which features are modulated separately in the frequency and spatial domains. The outputs of these dual branches are then fused through a multi-scale aggregation mechanism and a channel-gating module to generate cross-domain enhanced representations, enabling the network to more stably highlight aphid boundaries and body textures against complex field backgrounds.

Given an input feature map $X\in R^{C\times H\times W}$, we follow the spectral gating formulation of SpectFormer and learn, for each scale s∈S (e.g., S={30,60}), a complex-valued weighting map $W_{s}\in C^{H_{s}\times W_{s}}$:

(6) $$W_{s}=\alpha_{s}+j\beta_{s}$$

where $\alpha_{s},\beta_{s}\in R^{H_{s}\times W_{s}}$ denote the real and imaginary parts, respectively. Bilinear upsampling $u_{s}\left(\cdot \right)$ is applied to match the spatial resolution of *X*, yielding ${\tilde{\alpha }}_{s}=u_{s}\left(\alpha_{s}\right)\in R^{H\times W}$ and ${\tilde{\beta }}_{s}=u_{s}\left(\beta_{s}\right)\in R^{H\times W}$. These maps are then broadcast along the channel dimension to obtain tensors in $R^{C\times H\times W}$, which are used to modulate features at each scale.

In the frequency-enhancement branch, a unitary Fast Fourier Transform (FFT) [32] is first applied to *X*, denoted as $F:R^{C\times H\times W}\to C^{C\times H\times W}$, and its inverse counterpart (IFFT) $F^{-1}$ ensures energy consistency when mapping back to the spatial domain. The frequency-domain modulation at scale *s* is then given by

(7) $$X_{s}^{\left(\mathrm{freq}\right)}=F^{-1}\;\left(F\left(X\right)\;⊙\;\left({\tilde{\alpha }}_{s}+j{\tilde{\beta }}_{s}\right)\right)$$

where ⊙ denotes elementwise complex multiplication in the frequency domain. When ${\tilde{\alpha }}_{s}>1$ or $\left|{\tilde{\beta }}_{s}\right|$ increases at certain locations, the corresponding frequency bands are amplified (enhancing aphid edges and fine structural details); when ${\tilde{\alpha }}_{s}<1$, those bands are attenuated, which helps suppress reflection- or noise-dominated components.

At the same time, to avoid potential spatial artifacts caused by modulation exclusively in the frequency domain, FEAM introduces an additional spatial-domain pixel gate $X_{s}^{\left(\mathrm{sp}\right)}$. Specifically, the real part of the complex weight is used as a pixel-wise scaling factor:

(8) $$X_{s}^{\left(\mathrm{sp}\right)}={\tilde{\alpha }}_{s}⊙X$$

where ⊙ now denotes elementwise multiplication in the spatial domain. The broadcasted ${\tilde{\alpha }}_{s}$ ensures that all channels at the same spatial location are modulated consistently, directly enhancing responses in aphid regions while suppressing leaf-vein and shadow backgrounds. This design helps maintain boundary continuity and strengthens activation in dense aphid clusters or low-contrast areas.

The enhanced features from the dual branches are aggregated over all scales s∈S and fused via a simple multi-scale merge, computed as follows:

(9) $$Y=\frac{1}{\left|S\right|}\sum_{s\in S}\left(X_{s}^{\left(\mathrm{freq}\right)}+X_{s}^{\left(\mathrm{sp}\right)}\right)$$

where *Y* denotes the multi-scale, dual-domain fused feature map. Subsequently, a channel attention mechanism (Channel Gate) [33] is applied to adaptively reweight feature channels according to their task relevance:

(10) $$X_{\mathrm{final}}=Y⊙\mathrm{ChannelGate}\left(Y\right)$$

where ⊙ again indicates elementwise multiplication, and ChannelGate(·) outputs a channel-wise importance vector that highlights informative channels while suppressing less useful ones.

Compared with traditional methods that only run in the spatial domain or frequency domain, this dual-domain and multi-scale attention mechanism integrates complementary clues. The module enriches the feature representation and theoretically enhances the robustness of complex field conditions by capturing global low-frequency structures and local high-frequency details at the same time, and realizing precise frequency band modulation.

### 3.6. Experimental Settings

All experiments were conducted under the unified hardware and software environment summarized in Table 2. To maintain consistency across models, all networks were trained with the same basic optimisation hyperparameters, namely a batch size of 8 and 100 training epochs. To measure inference speed (FPS), we use a unified single-image benchmark for all models on the same GPU. FPS is computed as the number of processed images divided by the measured time, considering only the network forward pass and excluding data loading, resizing, visualization, and disk I/O. Under this protocol, we set the input resolution of the Aphid Cluster Segmentation dataset to 480×480, obtained by resizing the original 481×361 images, and the input resolution of the AphidSeg–Sorghum dataset to 1024×1024, obtained by resizing the original 1280×960 images; the corresponding FPS results are presented in the Section 4 along with the quantitative comparisons.

For optimization, we utilized the Adam optimizer to update network parameters. All models, including the baseline networks and the proposed FESW-UNet, were trained from scratch using the same data preprocessing pipeline and training–validation splits. This unified framework ensures that any observed performance differences result solely from model architectural variations.

### 3.7. Evaluation Indicators

Five common metrics, mean Intersection over Union (mIoU), mean Pixel Accuracy (mPA), Accuracy, mean F1-score (mF1), and Frames Per Second (FPS) [34], are selected for performance and efficiency assessment. They can be defined as follows:

(11) $$mIoU=\frac{1}{N}\sum_{i=1}^{N}\frac{TP_{i}}{TP_{i}+FP_{i}+FN_{i}}$$

(12) $$mPA=\frac{1}{N}\sum_{i=1}^{N}\frac{TP_{i}}{TP_{i}+FN_{i}}$$

(13) $$Accuracy=\frac{\sum_{i=1}^{N}TP_{i}}{\sum_{i=1}^{N}\left(TP_{i}+TN_{i}+FP_{i}+FN_{i}\right)}$$

(14) $$mF1=\frac{1}{N}\sum_{i=1}^{N}\frac{2TP_{i}}{2TP_{i}+FP_{i}+FN_{i}}$$

(15) $$FPS=\frac{1}{T_{inference}}$$

where *N* represents the number of classes. $TP_{i}$, $TN_{i}$, $FP_{i}$, and $FN_{i}$ denote the true positives, true negatives, false positives, and false negatives for class *i*, respectively. $T_{inference}$ represents the average inference time per image. These metrics follow standard definitions commonly used in semantic segmentation and are consistent with prior works.

## 4. Experimental Results

### 4.1. Model Comparative Experiments

In order to comprehensively evaluate the performance of the proposed FESW-UNet algorithm in aphid segmentation, we conducted a large number of comparative experiments on the Aphid Cluster Segmentation dataset. The dataset covers a variety of interference factors, including soil background, leaf texture changes and light changes, which preserve the controllability of laboratory conditions while approximating the complexity of real field environments and thus enable a reliable evaluation of model robustness. As presented in Table 3, FESW-UNet attains the highest overall performance among all comparison models, achieving an mIoU of 68.76%, mPA of 78.19%, Accuracy of 93.32%, and mF1 of 79.01%. Compared with the baseline UNet, which yielded an mIoU of 66.31%, mPA of 75.79%, Accuracy of 92.65%, and mF1 of 76.74%, the proposed model improves these metrics by 2.45, 2.40, 0.67, and 2.27 percentage points, respectively. Such improvements may be explained by the specified architectural considerations: the SimAM can improve a salient aphid-region representation and suppress background disturbances; the multi-scale dynamic weighting of EMA makes the model more capable of dealing with changes in scale in the field interactions; the HWD wavelet-based downsampling retains critical texture intelligence when downsampling resolution, thus preserving feature continuity; FEAM increases stability and preserves detail in challenging light–shadow interactions with the joint use of frequency and space domain data. Compared with other mainstream segmentation networks, including PSPNet, DeepLabv3+, Hernet, LR-ASPP, SegNet, UNet^2^, LinkNet, SegFormer, and FastSCNN, FESW-UNet demonstrates a clear advantage in most evaluation metrics and exhibits excellent consistency and boundary discrimination in scenes with strong reflections and shadow occlusion. Although the number of parameters increases (32.756 M against the 31.031 M of the baseline) slightly, the overcompared to is still relatively small and produces an acceptable balance between delivering quality and inference speed.

We further tested the FESW-UNet’s flexibility and cross-domain generalization capability in various settings. In particular, more comparative studies were done on the AphidSeg-Sorghum dataset. As summarized in Table 4, this dataset, which was filmed directly on actual sorghum fields, is more vigorous in illumination variation, texture-fertilized noise and morphology variance in aphid aggregates, and assesses more rigid robustness constraints to segmentation models. FESW-UNet achieves the best performance in all evaluation metrics, with an mIoU of 81.22%, mPA of 87.97%, Accuracy of 98.75%, and mF1 of 88.60%. Compared with the baseline UNet, which yielded an mIoU of 71.43%, mPA of 72.42%, Accuracy of 98.04%, and mF1 of 80.64%, these values correspond to improvements of 9.79, 10.55, 0.71, and 7.96 percentage points, respectively. FESW-UNet also consistently surpasses stronger competitors such as UNet^2^, LinkNet, and FastSCNN. The experiment results prove that FESW-UNet can be very advantageous due to the essential elements of the model: SimAM used to improve saliency, EMA used to model dynamism, HWD used to preserve the edges, and FEAM used to integrate the spatial-frequency features. These mechanisms ensure that the model can capture discriminative features and suppress background interference in textured, noisy environments. This is the result of the good segmentation performance of FESW-UNet, even when cross-domain is considered.

**Figure 8:**
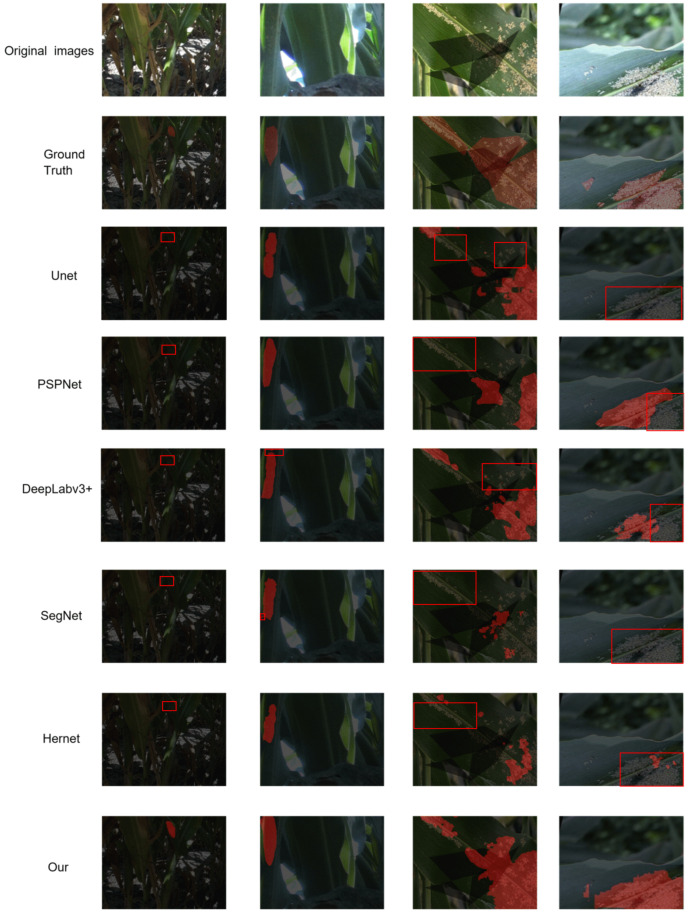
Qualitative comparison of segmentation results between FESW-UNet and other models on challenging field images. The red boxes highlight key areas where prediction errors occur.

To more intuitively showcase the segmentation capability of the proposed model under challenging field conditions, six representative segmentation networks (UNet, PSPNet, SegNet, Hernet, DeepLabv3+, and FESW-UNet) were selected for qualitative comparison across several typical scenarios, as illustrated in Figure 8. In the four displayed test images, the first two columns are from the Aphid Cluster Segmentation dataset, and the last two columns are from the AphidSeg-Sorghum dataset. All predictions were generated using models trained exclusively on the Aphid Cluster Segmentation dataset to evaluate cross-domain generalization rigorously. The visual results show that FESW-UNet can consistently achieve accurate target positioning and clear boundary division in an environment with uneven lighting, complex background textures and dense aphids. For example, in the first column of low-contrast and low-light scenes, FESW-UNet successfully reconstructed the complete aphid contour, while other models have obvious omissions. In the second column, most traditional models only detect a small part of the aphid area and fail to capture the complete target area. In contrast, FESW-UNet achieves better regional integrity and connectivity. The third list indicates that most other similar models will encounter problems of undersegmentation and oversegmentation in complex texture environments. At the same time, FESW-UNet makes full use of the advantages of FEAM’s frequency domain expansion and the edge retention characteristics of sampling under HWD to generate more coherent and stable segmentation diagrams. In column four, which is strongly illuminated and shaded by shadows, FESW-UNet still achieves good discrimination of aphids on the leaf surface, whereas PSPNet, DeepLabv3+, SegNet, and Hernet exhibit widespread cases of misclassification and irregular boundaries.

**Figure 9:**
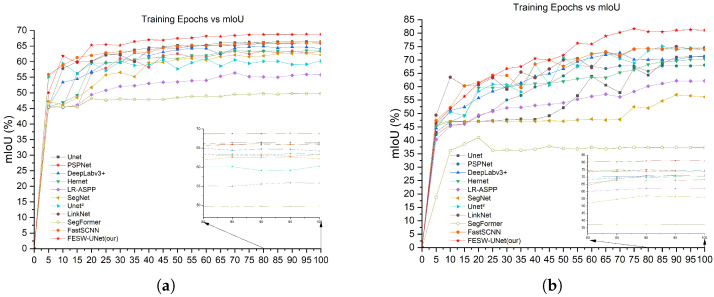
Comparison of mIoU performance curves during the training process: (**a**) Aphid Cluster Segmentation dataset; (**b**) AphidSeg-Sorghum dataset.

Furthermore, Figure 9 illustrates the mIoU comparisons between FESW-UNet and mainstream networks. Subfigure (a) corresponds to the Aphid Cluster Segmentation dataset. Subfigure (b) corresponds to the AphidSeg-Sorghum dataset. The curves demonstrate the stability and fast convergence speed of our model. These results further confirm the effectiveness and robustness of FESW-UNet. The results show that although FESW-UNet performs similarly to, or slightly below, other models in the early training stages, its performance steadily increases as training continues. The final curve converges at the highest level among all methods. This trend indicates that FESW-UNet can quickly acquire key discriminative patterns while suppressing noise, demonstrating efficient learning behavior and stable convergence. A reasonable explanation is that additional attention modules and frequency domain modules require a short preheating stage to learn meaningful weights, but then can provide stronger regularization and more expressive feature modeling. Due to its multi-scale and dual-domain feature enhancement strategy, FESW-UNet shows stronger generalization ability and a lower overfitting tendency. Overall, FESW-UNet not only achieves superior final segmentation accuracy compared with existing models but also demonstrates clear advantages in training stability and generalization performance.

Overall, the experimental results establish that FESW-UNet not only achieves better segmentation accuracy compared to all models but also provides a high level of real-time inference and robust performance of the tested model in complex field conditions. The model efficiently manages some difficult parameters, including the change of illumination, background noise, and texture interference, which yields more detailed, consistent, and stable aphid segmentation results. The results indicate that FESW-UNet provides an accurate, consistent and practically implementable technique to monitor the cases of aphids that are infesting sorghum in the real environment.

### 4.2. The Effect of Different Numbers of FEAM Modules on FESW-UNet

As illustrated in Figure 3, two Fourier-Enhanced Attention Modules (FEAMs) are progressively inserted along the UNet skip-connection pathway from deeper to shallower layers to form a fine-grained feature branch, thereby enhancing the model’s ability to capture high-frequency details and boundary structures. In the implementation phase, we instantiated a sequence of four FEAM blocks with predefined dual-scale settings spatial_scales = [30, 60], [60, 120], [120, 240], and [240, 480] for the four skip-connection levels, and the configurations “FEAM × 1”, “FEAM × 2”, “FEAM × 3”, and “FEAM × 4” correspond to activating the first one, two, three, and all four FEAM blocks in this list, respectively, so that each FEAM consistently operates on two spatial scales within its complex-modulation branch.

Since the number of stacked FEAM blocks may influence both the depth of feature extraction and the sensitivity to noise, we conducted an ablation study with different numbers of FEAM modules on the test split of the Aphid Cluster Segmentation dataset. As reported in Table 5, the model performs best when two FEAMs are employed, achieving an mIoU of 68.76%, mPA of 78.19%, Accuracy of 93.32%, mF1 of 79.01%, and an FPS of 254.780. This configuration thus offers an effective balance between segmentation accuracy and efficiency, without substantially increasing the parameter count (32.756 M).

Compared to the single FEAM configuration with an mIoU of 68.69% and mPA of 77.86%, the two-module configuration improves mIoU and mPA by 0.07 and 0.33 percentage points, respectively. This suggests that adding a second module further refines feature extraction and cross-scale integration. However, when the number of FEAM exceeds two (three or four modules), with mIoU dropping to 68.60% and 68.49%, we attribute this decline to increased network depth and redundancy, which may hinder gradient propagation and lead to overfitting given the current dataset size. Consequently, we adopted the two-module configuration for FESW-UNet, as it strikes the optimal balance between feature discrimination and inference speed.

### 4.3. Ablation Study

**Figure 10:**
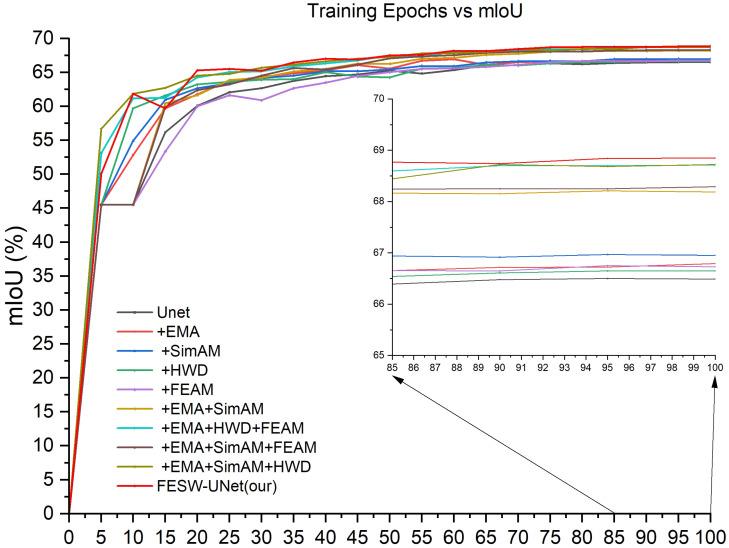
mIoU curves of the ablation experiment results for FESW-UNet.

To evaluate the contribution of each module, we conducted ablation experiments on the Aphid Cluster Segmentation dataset using UNet as the baseline (Table 6). The results show that adding the EMA module raised mIoU from 66.31% to 66.77% and mPA from 75.79% to 75.83%. These 0.46 and 0.04 percentage point gains demonstrate that EMA helps to enhance the global feature representation. Incorporating the lightweight SimAM mechanism (EMA + SimAM) resulted in mIoU and mPA values of 68.05% and 76.91%. Although somewhat different from using SimAM by itself, this combination is efficacious in improving the local feature activation and does not change the inference speed with respect to the baseline. Integrating the HWD module further enhanced performance, with the “EMA + SimAM + HWD” configuration achieving 68.62% mIoU and 77.67% mPA. The additional gains of 0.57% and 0.76% confirm that HWD preserves edge structures through wavelet decomposition, improving spatial detail without adding significant latency. Lastly, the addition of the FEAM completed the FESW-UNet architecture. FESW-UNet versus pre-FEAM arrangement upsurged the mIoU and mPA to 68.76% and 78.19%, respectively. Figure 10 shows in detail the mIoU variation curves for each ablation experiment on the training set of the Aphid Cluster Segmentation dataset. These results validate the benefit of using frequency-domain information to refine feature extraction and suppress noise.

In conclusion, FESW-UNet performed better than the baseline UNet in terms of all measures, achieving absolute gains of 2.45% in mIoU, 2.40% in mPA, 0.67% in Accuracy, and 2.27% in mF1. Notably, the accuracy improvement is accompanied by a substantial increase in inference throughput: FPS increased from 214.714 to 254.780, an improvement of 40.066 FPS (approximately 18.7%). The four modules are very complementary, where EMA deals with global semantics, SimAM deals with local regions, HWD deals with spatial structures, and FEAM deals with refining the details using frequency cues. Despite the added complexity, the total parameter count remains within the 31–33 million range, confirming that performance gains stem from efficient design rather than model inflation. Thus, FESW-UNet delivers high-precision segmentation and real-time processing, making it suitable for challenging field conditions.

## 5. Discussion

The FESW-UNet proposed in this paper is a dual-domain attention network, which aims to enhance the segmentation performance of sorghum aphids in complex field environments while maintaining high reasoning efficiency. By integrating SimAM, EMA, HWD, and FEAM into a UNet backbone, the model synergistically improves local saliency, multi-scale context modelling, edge preservation, and spectral feature refinement. Quantitative experiments on the Aphid Cluster Segmentation and AphidSeg-Sorghum datasets demonstrate that FESW-UNet significantly outperforms the baseline UNet and other representative networks (such as DeepLabv3+ and SegFormer) in terms of mIoU and mF1 scores. These results show that combining lightweight attention mechanisms with wavelet-based downsampling and Fourier-based spectral modulation is a highly effective strategy for handling small, densely clustered aphids under variable illumination. Specifically, SimAM enhances pixel-level distinctiveness by suppressing background noise; EMA improves the robustness of the model to scale changes; HWD retains high-frequency boundary information that is often lost in sampling under the standard; and FEAM uses spectrum to refine characteristics, so as to stabilize the segmentation results and protect them from complex blade textures and shadow transitions.

A comparative analysis reveals the structural advantages of FESW-UNet over mainstream architectures. Conventional models, such as DeepLabv3+, utilise dilated convolutions to expand the receptive field; although this method is effective for large objects, this approach often introduces gridding artefacts and leads to the loss of fine boundary details critical for segmenting minute insects. In contrast, by implementing HWD for downsampling, our model retains high-frequency edge data throughout the encoding process, preventing minute targets from being lost due to feature averaging. Furthermore, compared to Transformer-based models such as SegFormer, which excel at modelling long-range dependencies but may overlook local high-frequency textures, FESW-UNet explicitly enhances such textures in the frequency domain via FEAM. This dual-domain approach enables the model to distinguish the repetitive textural patterns of aphid colonies from complex leaf backgrounds more effectively than purely spatial-domain methods.

Although FESW-UNet has proven to provide performance advantages to the Aphid Cluster Segmentation dataset, as well as to the AphidSeg-Sorghum dataset, it is not devoid of a number of limitations. First, the present research is limited to RGB photographs of aphids on sorghum, and therefore, its generalizability to other crops, pest species, and sensing modalities has yet to be determined. By extending the framework to heterogeneous multi-source information (e.g., near-infrared, multispectral images), it is hoped that spectral confusion in mimetic backgrounds can be reduced, and further strengths can be obtained against diverse field conditions. Second, the reported inference speed is only assessed on a workstation level or class of GPUs, and the performance on embedded or resource-limited platforms has not been discussed. Future work will therefore focus on compressing and optimizing the model for deployment on ultra-lightweight drones and other edge devices, enabling large-scale real-time monitoring and sustainable, high-precision field management.

## 6. Conclusions

In this paper, FESW-UNet, a dual-domain attention network to detect sorghum aphids in complex field-like settings, is proposed. The model is built on top of a UNet backbone with elastic features of SimAM, EMA, HWD, and FEAM combined in one encoder–decoder system to mutually benefit local saliency, multi-scale context modeling, edge preservation, and spectral feature refinement. On the Aphid Cluster Segmentation dataset and the AphidSeg-Sorghum dataset, FESW-UNet achieves mIoU scores of 68.76% and 81.22% and mF1 scores of 79.01% and 88.60%, respectively, corresponding to improvements of 2.45 and 9.79 percentage points in mIoU and 2.27 and 7.96 percentage points in mF1 over the baseline UNet. Comparative experiments with various prominent segmentation models and ablation studies of the four primary modules all show that FESW-UNet has a good trade-off between segmentation quality and high reasoning efficiency. Overall, the proposed network offers a viable foundation for automated aphid surveillance of sorghum and has the potential to support precision pest management in intelligent agriculture when combined with appropriate sensing and deployment platforms.

## Figures and Tables

**Table 1:** Summary of the datasets used in this study.

Dataset	Source	No. of Images	Annotation	Augmentation	Split
Aphid Cluster Segmentation dataset	Rahman et al. [27]	7720	Pixel-level aphid regions	None (original images)	70%/30% (train/test)
AphidSeg-Sorghum dataset	This study	1224	Pixel-level aphid regions	Flip, brightness adjustment, noise injection	70%/30% (train/test)

**Table 2:** Training environment settings.

Category	Component	Specification
Hardware environment	CPU	Intel(R) Xeon(R) Silver 4210R @ 2.40 GHz (Intel, Santa Clara, CA, USA)
	GPU	NVIDIA GeForce RTX 3080 (10 GB VRAM) (Nvidia, Santa Clara, CA, USA)
	RAM	32 GB
Software environment	Operating system	Ubuntu 20.04.6
	Deep learning framework	PyTorch 2.0.1
	Python	3.8
	CUDA Toolkit	CUDA 12.2

**Table 3:** Comparison results of different models on the Aphid Cluster Segmentation dataset.

Model	mIoU (%)	mPA (%)	Accuracy (%)	mF1 (%)	FPS	Para (M)
UNet	66.31	75.79	92.65	76.74	214.714	31.031
PSPNet [35]	63.20	68.92	92.99	73.31	175.847	2.381
DeepLabv3+ [36]	63.52	69.91	92.83	73.71	272.790	5.813
Hernet [37]	63.63	69.65	92.98	73.79	266.132	9.395
LR-ASPP [38]	55.80	60.95	91.40	64.61	99.362	3.218
SegNet [39]	62.68	71.69	91.74	73.11	242.162	29.444
UNet^2^ [40]	61.62	68.67	92.13	71.77	241.693	9.163
LinkNet [41]	65.97	74.90	92.71	76.38	234.002	11.534
SegFormer [42]	49.73	55.60	88.78	56.76	241.295	**0.395**
FastSCNN [43]	66.23	76.75	92.38	76.73	243.574	1.136
**FESW-UNet (ours)**	**68.76**	**78.19**	**93.32**	**79.01**	**254.780**	32.756

Note: Bold values indicate the best result in each column.

**Table 4:** Comparison results of different models on the AphidSeg-Sorghum dataset.

Model	mIoU (%)	mPA (%)	Accuracy (%)	mF1 (%)	FPS	Para (M)
UNet	71.43	77.42	98.04	80.64	75.697	31.031
PSPNet [35]	69.85	75.30	97.82	78.70	73.850	2.381
DeepLabv3+ [36]	70.12	76.08	97.90	79.08	**78.460**	5.813
Hernet [37]	70.25	75.86	97.93	79.23	76.310	9.395
LR-ASPP [38]	61.20	66.95	96.90	68.43	70.120	3.218
SegNet [39]	55.23	59.08	96.57	61.33	74.200	29.444
UNet^2^ [40]	74.55	80.05	98.32	83.26	74.630	9.163
LinkNet [41]	74.76	82.25	98.22	83.46	75.050	11.534
SegFormer [42]	37.14	45.13	72.77	43.69	74.500	**0.395**
FastSCNN [43]	73.67	79.50	98.23	82.50	76.200	1.136
**FESW-UNet (ours)**	**81.22**	**87.97**	**98.75**	**88.60**	75.348	32.76

Note: Bold values indicate the best result in each column.

**Table 5:** Experimental results using different numbers of FEAM modules on the Aphid Cluster Segmentation dataset.

Model	mIoU (%)	mPA (%)	Accuracy (%)	mF1 (%)	FPS	Para (M)
UNet-SimAM-EMA-HWD-FEAM × 1	68.69	77.86	93.36	78.93	254.127	**32.723**
UNet-SimAM-EMA-HWD-FEAM × 2	**68.76**	**78.19**	93.32	**79.01**	**254.780**	32.756
UNet-SimAM-EMA-HWD-FEAM × 3	68.60	77.44	**93.40**	78.83	251.407	32.765
UNet-SimAM-EMA-HWD-FEAM × 4	68.49	77.97	93.24	78.76	250.062	32.767

Note: Bold values indicate the best result in each column.

**Table 6:** Ablation studies on the Aphid Cluster Segmentation dataset.

Model	mIoU (%)	mPA (%)	Accuracy (%)	mF1 (%)	FPS	Para (M)
UNet	66.31	75.79	92.65	76.74	214.714	**31.031**
+EMA	66.77	75.83	92.96	77.23	219.162	31.196
+SimAM	66.75	75.71	92.90	77.12	236.645	31.031
+HWD	66.61	75.98	92.77	77.02	236.174	32.427
+FEAM	66.75	76.29	92.76	77.16	236.874	31.197
+EMA + SimAM	68.05	76.91	93.26	78.33	240.511	31.196
+EMA + HWD + FEAM	68.72	78.07	93.32	78.96	238.593	32.756
+EMA + SimAM + FEAM	68.03	77.01	93.23	78.32	239.752	31.361
+EMA + SimAM + HWD	68.62	77.67	**93.36**	78.85	242.626	32.592
**FESW-UNet**	**68.76**	**78.19**	93.32	**79.01**	**254.780**	32.756

Note: Bold values indicate the best result in each column.

## Data Availability

The datasets can be provided by the corresponding author upon reasonable request.
